# Complete vs. incomplete percutaneous revascularization in patients with chronic total coronary artery occlusion

**DOI:** 10.3389/fcvm.2024.1443258

**Published:** 2024-07-23

**Authors:** Luis Carlos Maestre-Luque, Rafael Gonzalez-Manzanares, Javier Suárez de Lezo, Francisco Hidalgo, Lucas Barreiro-Mesa, Jaime de Juan, Ignacio Gallo, Jorge Perea, Marco Alvarado, Miguel Romero, Soledad Ojeda, Manuel Pan

**Affiliations:** ^1^ Department of Cardiology, Reina Sofia University Hospital, Cordoba, Spain; ^2^ Maimonides Biomedical Research Institute of Cordoba (IMIBIC), Cordoba, Spain; ^3^ Centro de Investigación Biomédica en Red Enfermedades Cardiovasculares (CIBERCV), Madrid, Spain; ^4^ Department of Cardiology, Virgen del Rocío University Hospital, Sevilla, Spain; ^5^ Department of Medicine, University of Cordoba, Cordoba, Spain

**Keywords:** chronic total occlusion, percutaneous coronary intervention, coronary artery disease, major adverse cardiovascular events, myocardial infarction

## Abstract

**Introduction:**

There is current controversy surrounding the benefits of percutaneous coronary intervention (PCI) of chronic total coronary occlusions (CTO). We aimed to evaluate the impact of complete percutaneous revascularization on major adverse cardiovascular events (MACE) in patients with CTO.

**Methods:**

A retrospective observational study was conducted of consecutive patients referred for invasive coronary angiography at a single center between January 2018 and December 2019 and at least a CTO. The patients were divided into two groups according to the result of the procedure: complete revascularization of CTO (CR-CTO) versus incomplete revascularization (ICR-CTO) (patients with at least one non-recanalized CTO). Short- and mid-term clinical outcomes were evaluated. The primary endpoint was a composite of MACE that included all-cause death, non-fatal myocardial infarction, non-fatal stroke, or unplanned revascularization.

**Results:**

In total, 359 patients with CTO were included. The median age was 68 years [interquartile range (IQR) 60–77 years], 66 (18%) were women and 169 (47.3%) had diabetes mellitus. In all, 167 (46.5%) patients received complete revascularization. After a median follow-up of 42 months (IQR 46–50 months), the primary endpoint occurred in 39 (23.4%) patients in the CR-CTO group and in 75 (39.1%) in the ICR-CTO group (HR 0.50, 95% CI 0.34–0.74; *p* < 0.001). This association remained significant in an inverse probability weighted model considering prognostic factors (adjusted HR 0.61, 95% CI 0.41–0.92; *p* = 0.018) and was driven by lower rates of all-cause death (adjusted OR 0.50, 95% CI 0.23–0.84; *p* = 0.01).

**Conclusions:**

Complete revascularization of CTO was associated with a lower risk of MACE in the midterm follow up.

## 1 Introduction

Chronic total occlusions (CTO) are a relatively common finding in patients with coronary artery disease (CAD). The prevalence of these lesions can reach up to 15%–25% of patients with stable angina pectoris (1), and up to 10%–15% of those presenting with ST-segment elevation myocardial infarction (STEMI) (2, 3). The presence of a CTO confers a worse prognosis for patients in terms of quality of life (4) and global mortality (5).

Percutaneous coronary intervention of chronic total occlusions (CTO-PCI) is a technically demanding procedure that requires trained and experienced professionals. Nevertheless, the success rate of CTO recanalization has improved in recent years because of the development of new techniques, advances in devices, and increasing experience. Recent prospective registries report procedural success rates in the range of 75%–90% (6–9).

There is controversial evidence on the benefits of CTO-PCI. Large observational studies and randomized control trials (RCT) have shown a positive effect of CTO-PCI on health status (10), left ventricular ejection fraction (LVEF) (11, 12), burden of ventricular arrhythmia (13), and overall survival (14). However, there are neutral or negative clinical trials that did not show evidence of LVEF recovery (15, 16) or a treatment effect on MACE (17).

The combination of the procedural complexity and the lack of robust evidence supporting a beneficial prognostic effect of CTO-PCI poses a major barrier for the widespread implementation of CTO recanalization. In fact, only 4%–10% of PCIs aim for CTO revascularization (8, 18).

This study sought to evaluate the association between complete or incomplete revascularization of CTOs and mid-term clinical outcomes in a high-volume center.

## 2 Materials and methods

### 2.1 Study design and population

An observational, longitudinal, retrospective study was conducted of all consecutive patients discharged with a diagnosis of CTO at a single center between January 2018 and December 2019. The inclusion criteria were as follows: (i) diagnosis of chronic total occlusion involving at least one of the three main genuine coronary vessels [left anterior descending coronary artery (LAD), left circumflex coronary artery (LCA), right coronary artery (RCA)]; and (ii) age equal to or greater than 18 years at diagnosis. The exclusion criteria were as follows: (i) surgical treatment of chronic total occlusion; (ii) in-hospital death during the same hospitalization when CTO was diagnosed; and (iii) patients' habitual residence located out of the region. Patients were divided into two groups according to the result of the percutaneous procedure: complete revascularization of CTO (CR-CTO) (patients with all CTO lesions located on the three main coronary vessels being revascularized) versus incomplete revascularization of CTO (ICR-CTO) (patients with at least one non-recanalized CTO located on one of the three main coronary arteries). This study was conducted according to the Declaration of Helsinki and was approved by the local clinical research ethics committee. The data were anonymized, and confidentiality was preserved, in accordance with the Regulation 2016/679—Protection of natural persons with regard to the processing of personal data and on the free movement of such data, and repealing Directive 95/46/EC (General Data Protection Regulation).

### 2.2 Procedures and medications

CTO-PCI indication was made by a multidisciplinary team comprising clinical cardiologists, interventional cardiologists, and cardiac surgeons considering the risks and benefits of the intervention, technical aspects of the lesions, and patient preferences. The decisions were in accordance with the clinical guidelines (19, 20) and established standards of practice (21). The extent of CAD and the SYNTAX score (Synergy between PCI with TAXUS and Cardiac Surgery) were assessed at the angiographic laboratory by interventional cardiologists. Medical treatment was optimized by the clinicians after routine clinical practice (20). Clinical and procedural data, treatment at discharge, and outcomes during the follow-up were reviewed through electronic health records.

### 2.3 Definitions

Chronic total occlusion was defined as angiographically proven anterograde flow obstruction of a coronary artery, known, or suspected to have lasted >3 months [with Thrombolysis In Myocardial Infarction (TIMI) flow = 0] (22), based on the patient's history. When no definite evidence of occlusion duration existed, the diagnosis of CTO was made based on angiographic morphology by at least two experienced interventional cardiologists. Successful revascularization was defined as angiographic final residual stenosis <20% by visual estimation and TIMI flow grade 3 after CTO recanalization.

Myocardial infarction (MI) was defined following the universal definition endorsed by the European Society of Cardiology (23). Worsening heart failure (WHF) was defined as the need for increasing diuretic dose or hospitalization for intravenous therapy. Clinically relevant bleeding was defined as a bleeding event type 2, 3, or 5 according to the Bleeding Academic Research Consortium (BARC) (24).

### 2.4 Endpoints

The primary endpoint was a composite of MACE, based on *Academic Research Consortium-2* criteria (25), which included: all-cause death, non-fatal myocardial infarction, non-fatal stroke, or unplanned revascularization.

Secondary endpoints were the individual components of the primary endpoint, worsening heart failure, visit to the emergency department or unplanned hospitalization due to chest pain, and clinically relevant bleeding.

### 2.5 Statistical analysis

Data are expressed as absolute and percent frequency in the case of qualitative variables. Quantitative variables are expressed as mean ± standard deviation or median (interquartile range), depending on variable distribution. The normality of distribution was assessed using the Shapiro–Wilk test and Q–Q plots. Between-group comparisons were performed using the Student’s *t*-test or its non-parametric equivalent, the Mann–Whitney *U*-test, for continuous variables, and the chi-square test or Fisher's exact test for categorical variables. To evaluate the risk of MACE, time-to-event analyses were conducted using Kaplan–Meier and Cox proportional hazards methods. Logistic regression models were fitted to calculate the odds ratio for the secondary endpoints. All the models were adjusted by inverse probability of treatment weighting (IPTW) (26). Propensity scores were calculated using a logistic regression model that included those covariates with a prognostic impact according to previous literature: age, sex, glomerular filtration, diabetes mellitus, LVEF, localization of CTO, and extent of CAD. A standardized mean difference (SMD) of <10% was considered to indicate good balance. Confidence intervals for the IPTW coefficients were obtained using robust sandwich-type variance estimators (27). All tests were two-tailed and were considered significant when *p* < 0.05. Statistical analyses were performed using R software (version 4.0.3; R Foundation for Statistical Computing, Austria).

## 3 Results

### 3.1 Baseline clinical and angiographic characteristics

A total of 359 patients were included. The median age was 68 years (IQR 60–77), 66 (18%) patients were women, and 169 (47.3%) had diabetes mellitus. The mean LVEF was 55% ± 13%. Most patients were symptomatic at diagnosis (80% chest pain, 20.6% heart failure). Complete revascularization of CTO was performed in 167 (46.5%) patients (CR-CTO group), whereas 192 (53.5%) patients had at least a non-revascularized CTO (ICR-CTO group). CR-CTO patients were younger [66 (IQR 59–74) vs. 70 (IQR 61–79) years; *p* < 0.001] and had greater glomerular filtration rate [95.9 (IQR 70.9–121.5) vs. 88.8 (IQR 56.4–117.1) mL/min/1.73 m^2^; *p* 0.036]. They were more likely to have a history of coronary artery disease [78 (46.7%) vs. 68 (36.0%); *p* = 0.040] and prior PCI [67 (52.8%) vs. 43 (35.8%); *p* = 0.007], but they had lower rates of prior coronary artery bypass grafting (CABG) [2 (1.7%) vs. 10 (8.4%); *p* = 0.016]. This group also had more evidence of myocardial ischemia using non-invasive tests [74.0 (44.8%) vs. 45 (24.7%); *p* < 0.001]. The main clinical characteristics are summarized in Table 1.

**Table 1:** Baseline clinical characteristics.

	CR-CTO *N* = 167	ICR-CTO *N* = 192	*p*
Age (years)	66 (59–74)	70 (61–92)	<0.001
Female sex	31 (18.6)	35 (18.2)	0.935
Obesity	85 (52)	100 (61)	0.120
Diabetes	94 (49.7)	75 (44.9)	0.363
Hypertension	121 (72.5)	147 (77.8)	0.245
Hyperlipidemia	92 (55.1)	113 (59.8)	0.371
Current or former smoker	49 (29.5)	41 (21.8)	0.096
EGFR (ml/min/1.73 m^2^)	96 (71–121)	89 (65–117)	0.036
Chronic kidney disease	26 (16.2)	45 (26.8)	0.021
Atrial fibrillation	18 (11)	30 (16)	0.168
LVEF (%)	57.1 (46–68)	53.8 (40–64)	0.082
LVEF <40%	19 (17.9)	33 (23.6)	0.283
History of stroke	12 (7.3)	14 (7.5)	0.940
History of PVD	22 (13.3)	28 (15.1)	0.629
History of CAD	78 (46.7)	68 (36)	0.040
Multivessel CAD	75 (44.9)	128 (66.7)	<0.001
Prior PCI	67 (40.1)	43 (22.4)	0.007
Prior CABG	2 (1.2)	10 (5.2)	0.016
Clinical presentation			
Chest pain	135 (81.8)	145 (78.4)	0.422
Dyspnea	39 (23.6)	55 (30.2)	0.168
Heart failure	29 (17.7)	45 (25)	0.099
Evidence of ischemia	74 (44.8)	45 (24.7)	<0.001

CABG, coronary artery bypass grafting; CAD, coronary artery disease; EGFR, estimated glomerular filtration rate; LVEF, left ventricular ejection fraction; PAD, peripheral vascular disease; PCI, percutaneous coronary intervention. Data are expressed as absolute and percent frequency for qualitative variables Quantitative variables are expressed as mean ± standard deviation.

There were 407 angiographically diagnosed CTO in 359 patients, 211 (51.8%) located in the RCA, 103 (25.3%) in the LCA, and 93 (22.9%) in the LAD. The mean SYNTAX score was 21.4 (95% CI 17.5–25.8), without differences between groups. Technical success was achieved in 203 (89%) out of a total of 228 attempted lesions. In those patients with incomplete revascularization, 234 CTO were diagnosed and 179 (76.5%) were left to pharmacological treatment. This group carried a greater rate of multivessel coronary artery disease (including CTO and non-CTO lesions) [128 (66.7%) vs. 75 (44.9%); *p* < 0.001]. The main angiographic and procedure characteristics are provided in Table 2.

**Table 2:** Angiographic characteristics.

	CR-CTO *N* = 167	ICR-CTO *N* = 192	*p*
Total number of CTO	173	234	
SYNTAX score	20.2 (15.3–24.7)	22.5 (17.5–26.8)	0.143
LAD CTO	52 (30)	41 (17.5)	0.035
Medical treatment	0 (0)	27 (66)	
PCI success	52 (100)	10 (24)	
PCI failure	0 (0)	4 (10)	
LCA CTO	30 (17.3)	73 (31.2)	<0.001
Medical treatment	0 (0)	56 (76.7)	
PCI success	30 (100)	8 (11)	
PCI failure	0 (0)	9 (12.3)	
RCA CTO	91 (52.7)	120 (51.3)	0.124
Medical treatment	0 (0)	96 (80)	
PCI success	91 (100)	12 (10)	
PCI failure	0 (0)	12 (10)	

CTO, chronic total occlusion; CR-CTO, complete revascularized CTO; ICR, incomplete revascularized CTO; LAD, left anterior descending coronary artery; LCX, left circumflex coronary artery; RCA, right coronary artery; PCI, percutaneous coronary intervention. Percentages are calculated over the total number of CTO.

### 3.2 Clinical outcomes

**Figure 1:**
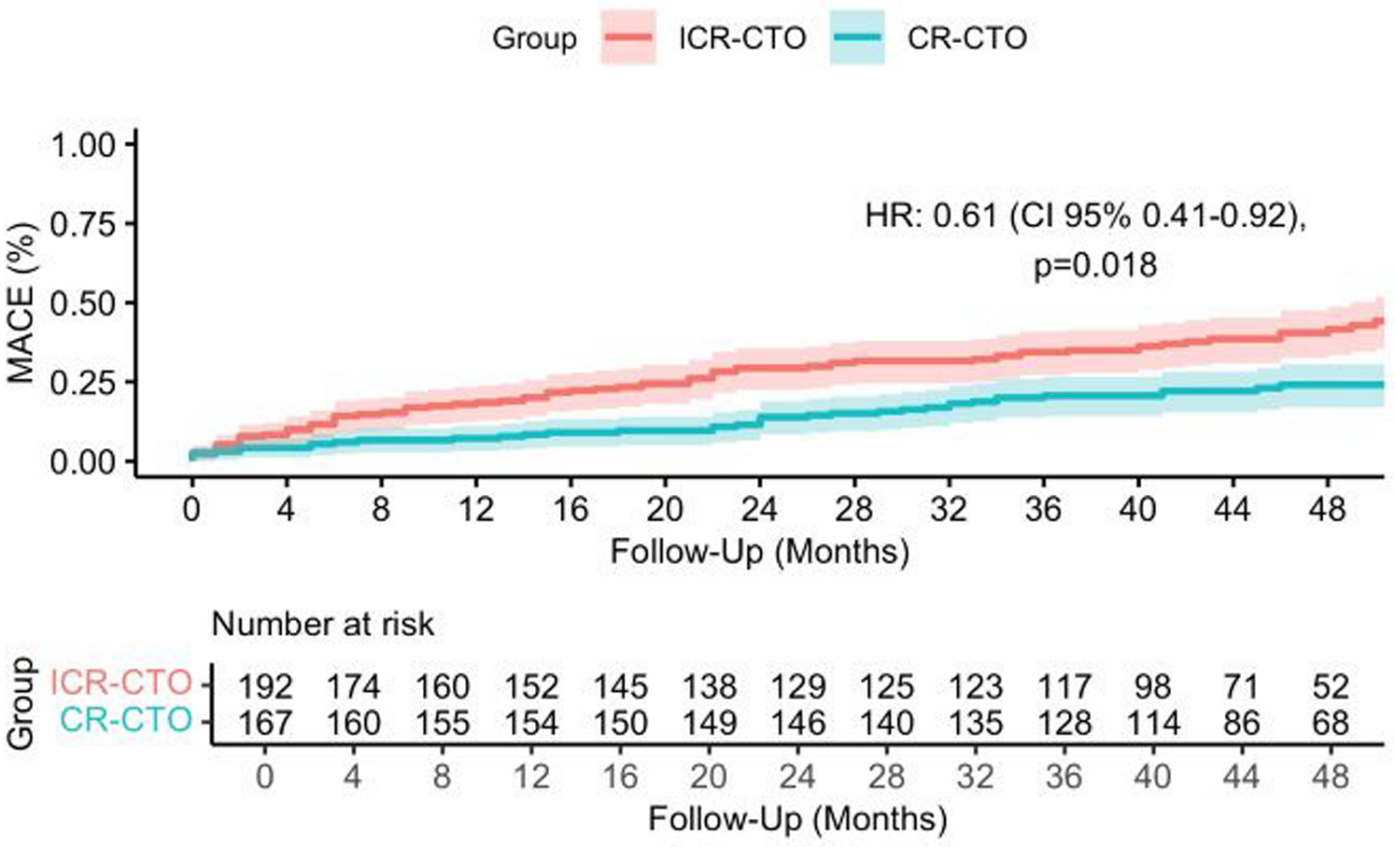
Kaplan–Meier curve for major adverse cardiovascular events. CR-CTO, complete revascularized chronic total occlusion; ICR-CTO, incomplete revascularized chronic total occlusion.

**Figure 2:**
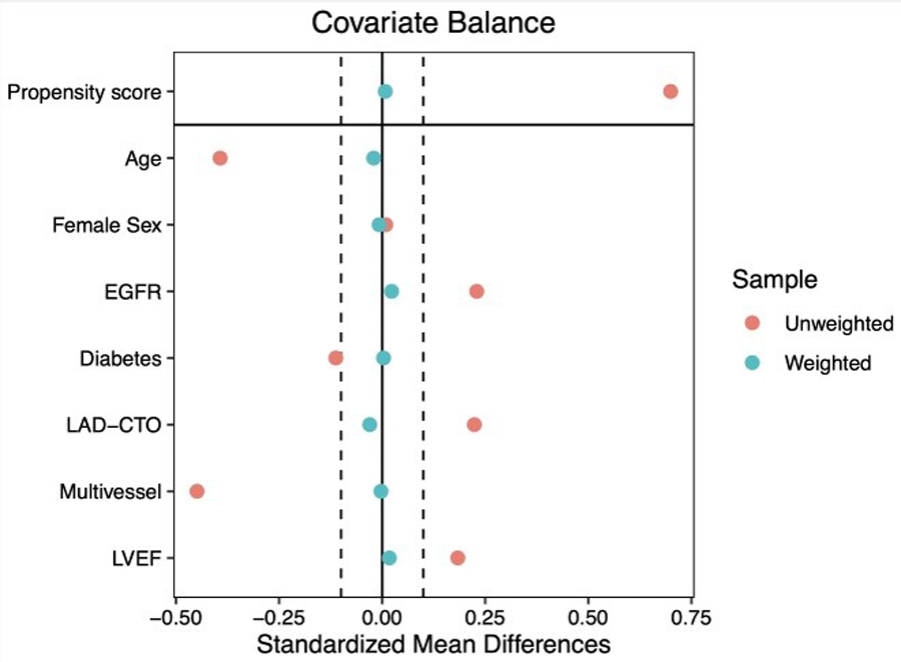
Covariate balance in the unweighted and weighted population. EGFR, estimated glomerular filtration rate; LAD-CTO, left anterior descending chronic total occlusion; LVEF, left ventricular ejection fraction.

After a median follow-up of 42 months (IQR 46–50 months), the primary endpoint occurred in 39 (23.4%) patients in the CR-CTO group and in 75 (39.1%) patients belonging to the ICR-CTO group (HR 0.50, 95% CI 0.34–0.74, *p* < 0.001) (Figure 1). To adjust for prognostic relevant confounders, we fitted an IPTW adjusted Cox model. The covariables included in the model showed an excellent balance with SMD <10% (Figure 2). In the IPTW adjusted Cox's model, the association remained significant [adjusted HR (HRadj) 0.61, 95% CI 0.41–0.92, *p* = 0.018]. Complete revascularization was also associated with a lower rate of all-cause death [adjusted OR (ORadj) 0.5, 95% CI 0.3–0.84, *p* = 0.01]. In the unadjusted analysis, the CR-CTO group showed a lower rate of non-fatal MI (OR 0.40, 95% CI 0.17–0.92, *p* = 0.038) and WHF (OR 0.47, 95% CI 0.27–0.81, *p* = 0.007). However, in the adjusted IPTW analysis, there was a numerical but not significant difference in the risk of non-fatal MI (ORadj 0.53, 95% CI 0.21–1.2, *p* = 0.146) and WHF (ORadj 0.62, 95% CI 0.36–1.06, *p* = 0.088) events. There were no differences between the groups in the rate of non-fatal stroke (ORadj 0.7, 95% CI 0.25–2.37, *p* = 0.693), unplanned revascularization (ORadj 1.4, 95% CI 0.6–3.2, *p* = 0.425), visit to the emergency department or unplanned hospitalization due to chest pain (ORadj 0.93, 95% CI 0.59–1.47, *p* = 0.764), and clinically relevant bleeding (ORadj 0.78, 95% CI 0.45–1.34, *p* = 0.375).

The results of the analyses of primary and secondary endpoints in the unweighted and weighted population are provided in Table 3.

**Table 3:** Primary and secondary endpoints: results of the analyses in the unweighted and weighted population.

	CR-CTO *N* = 167	ICR-CTO *N* = 192	Unadjusted model		IPTW adjusted model	
	*n* (%)	*n* (%)	HR (CI 95%)	*p*	HR (CI 95%)	*p*
Primary endpoint						
MACE	39 (23.4)	75 (39.1)	0.50 (0.34–0.74)	<0.001	0.61 (0.41–0.92)	0.018
Secondary endpoints						
All-cause death	24 (14.4)	62 (32.3)	0.35 (0.20–0.59)	<0.001	0.5 (0.3–0.84)	0.010
Non-fatal MI	8 (5)	21 (11)	0.40 (0.17–0.92)	0.038	0.53 (0.21–1.2)	0.146
Non-fatal stroke	6 (3.6)	8 (4.2)	0.85 (0.28–2.51)	0.780	0.7 (0.25–2.37)	0.693
Unplanned revascularization	13 (7.8)	12 (6.2)	1.26 (0.55–2.89)	0.570	1.4 (0.6–3.2)	0.425
Worsening heart failure	24 (14.4)	50 (26)	0.47 (0.27–0.81)	0.007	0.62 (0.36–1.06)	0.088
Chest pain (emergency/hospital)	46 (27)	54 (28)	0.97 (0.61- 1.54)	0.903	0.93 (0.59–1.47)	0.764
Clinically relevant bleeding	25 (15)	38 (19)	0.71 (0.41–1.24)	0.232	0.78 (0.45–1.34)	0.375

CI, confidence interval; CR-CTO, complete revascularized chronic total occlusion; ICR-CTO, incomplete revascularized chronic total occlusion; IPTW, inverse probability of treatment weighting; HR, hazard ratio; MI, myocardial infarction; OR, odds ratio. The covariates included in the IPTW adjusted model were age, sex, glomerular filtration, diabetes mellitus, heart failure, localization of CTO, extent of CAD.

## 4 Discussion

In this study, we found that complete CTO percutaneous revascularization compared to incomplete revascularization (a) was associated with lower overall risk of MACE and that (b) this lower risk was driven by overall mortality.

Most baseline characteristics of patients were analogous to the studies involving CTO: multiple cardiovascular risk factors, preserved LVEF, complex CAD, and previous history of revascularization by PCI or CABG. However, the median age was slightly higher than the reported by other observational works (7, 8, 28). ICR-CTO was mostly a consequence of the operator's decision to not attempt CTO-PCI. It might be explained by the presence of some factors in this group that increased the procedural risk: older age, lower rates of glomerular filtration, more severe coronary artery disease, and greater burden of previous CABG. In particular, renal function is an important factor during decision-making (29). The association between complete revascularization and a lower risk of MACE was present after adjusting for relevant prognostic factors that were included in the IPWT adjusted Cox model. Patients in the CR-CTO group had lower overall mortality, suggesting a beneficial effect of complete revascularization of CTO. We did not find differences in non-fatal MI or WHF between groups in the adjusted analysis. The similar rates of non-fatal MI could be explained by the fact that the risk of subsequent MI might be driven by the progression of mild atherosclerotic plaques and the presence of new unstable non-CTO lesions, rather than by previous non-revascularized CTO. Concerning WHF, some factors might explain our findings: there were no differences in previous history of heart failure or LVEF between groups and most patients had preserved ejection fraction, which could limit the benefit of CTO-PCI in terms of LVEF recovery. Interestingly, we did not find differences in the rates of major bleeding events, which might be considered a potential falsification endpoint supporting the robustness of the adjustment (30). The rate of successful procedures (90%) is in line with the results reported in high-volume and experienced centers (8, 9, 31).

There have been controversial and conflicting findings about CTO-PCI in observational studies and RCTs. Regarding symptoms and quality of life, the revascularization of CTO has proved to be beneficial compared to optimal medical treatment in the EURO-CTO trial (10). The IMPACTOR-CTO trial showed a reduction in inducible ischemia burden (32), measured by magnetic resonance image (MRI), with CTO-PCI. Concerning LVEF and cardiac remodeling, some observational studies have reported an improvement after CTO recanalization (11, 12), the EXPLORE trial suggested a beneficial effect only for patients with CTO-PCI targeting LAD (15), and the REVASC trial did not find a benefit in terms of LVEF recovery (16). In relation to hard endpoints, the two largest RCTs so far have shown no impact of CTO recanalization on MACE (10, 17), with some limitations to point out. First, the EURO-CTO was not designed to test hard endpoints and the follow-up period was short. Second, the DECISION-CTO had high rates of crossover between treatment groups, they evaluated a combined strategy of PCI both for CTO and non-CTO lesions, and the sample size was smaller than planned, which reduced the power to test MACE. On the contrary, several non-randomized studies have suggested a positive effect of CTO-PCI on hard endpoints. Multicenter prospective study IRCTO showed a reduction of MACE and cardiovascular death in CTO-PCI patients during a short-term follow-up (33), and other observational studies obtained similar results (34, 35). The ERCTO prospective registry revealed lower rates of MACE, including cardiac death, myocardial infarction, and non-planned revascularization, in completely retrograde revascularized patients (36). Azzalini et al. suggested that even a mild degree of incomplete revascularization in patients with CTO (residual SYNTAX score between 1 and 8) is associated with a higher incidence of MACE on long-term follow-up (37). In addition, several meta-analyses of observational studies have reported a beneficial effect of CTO-PCI. Some of them showed a reduction of MACE for complete revascularization of CTO versus optimal medical treatment alone (28–39). Others have compared successful versus unsuccessful revascularization of CTO and obtained a significant benefit of complete recanalization on long-term MACE and reduced needs for subsequent CABG (14, 40, 41).

Our results are in line with previous large observational studies and multicenter registries and point toward a potential benefit of complete CTO revascularization in terms of MACE. Potential explanations for our findings include a reduction in ischemic burden and the risk for arrhythmias, the better outcomes in case of an acute coronary syndrome (the area at risk of necrosis is higher for incomplete revascularized CTO patients when an atherosclerotic plaque is unstable in a non-CTO vessel) or the improvement in left ventricular function and cardiac remodeling.

Considering the continuous technical advancements and improvements in CTO-PCI success rates, the revascularization of CTOs seems an appealing option that potentially leads to an added prognostic benefit, especially in symptomatic patients. It should be acknowledged that non-randomized studies are necessary to continue building a body of evidence in CTO-PCI, where well-designed and powered RCTs are lacking. Two ongoing randomized control trials (NOBLE-CTO and ISCHEMIA-CTO) could shed light on the prognostic value of CTO-PCI.

Our research has some limitations. The observational nature of our study provides only associative evidence, and we cannot rule out the presence of residual confounding factors due to the lack of randomization. There is also potential for survival or selection bias (unfavorable patients' characteristics and a more complex anatomy might have influenced the decision for a conservative treatment). To minimize those issues, we conducted a propensity score-based analysis accounting for prognostic factors. Furthermore, the single-center design of the study might limit the generalization of our results to other centers depending on the operator's experience. Finally, since the SARS-Cov-2 pandemic occurred during the study period, our results might have been influenced by changes in the healthcare system's accessibility. The lower accessibility to hospitals during the pandemic might have resulted in an underestimation of cardiovascular events.

In conclusion, patients with CTO who received complete revascularization had a lower midterm risk of MACE, mainly driven by a reduction in the rates of all-cause death. These results suggest a potential benefit of PCI-CTO and supply real-world data for routine practice that could help to guide clinical decision-making. More randomized control trials are needed to generate robust evidence on this topic.

## Data Availability

The raw data supporting the conclusions of this article will be made available by the authors, without undue reservation.
